# Dichotomy between the transcriptomic landscape of naturally versus accelerated aged murine hearts

**DOI:** 10.1038/s41598-020-65115-9

**Published:** 2020-05-18

**Authors:** Federica De Majo, Jana-Charlotte Hegenbarth, Frank Rühle, Christian Bär, Thomas Thum, Martine de Boer, Dirk J. Duncker, Blanche Schroen, Anne-Sophie Armand, Monika Stoll, Leon J. De Windt

**Affiliations:** 1 Department of Molecular Genetics, Faculty of Science and Engineering; Maastricht University, Maastricht, The Netherlands; 2 CARIM School for Cardiovascular Diseases, Faculty of Health, Medicine and Life Sciences; Maastricht University, Maastricht, The Netherlands; 3 Bioinformatics Core Facility, Institute of Molecular Biology (IMB), Mainz, Germany; 4 Department of Genetic Epidemiology, Institute of Human Genetics, University Hospital Münster, Münster, Germany; 5 Institute of Molecular and Translational Therapeutic Strategies (IMTTS), Hannover Medical School, Hannover, Germany; 6 REBIRTH Excellence Cluster, Hannover Medical School, Hannover, Germany; 7 Division of Experimental Cardiology, Department of Cardiology, Thoraxcenter, Erasmus MC, University Medical Center Rotterdam, Rotterdam, The Netherlands; 8 Institut Necker Enfants Malades, Inserm U1151, Paris, France; Universite Paris Descartes, Sorbonne Paris Cite, Paris, France; 9 Department of Biochemistry, Cardiovascular Research Institute Maastricht, Maastricht University, Maastricht, The Netherlands

**Keywords:** Gene regulatory networks, Transcriptomics

## Abstract

We investigated the transcriptomic landscape of the murine myocardium along the course of natural aging and in three distinct mouse models of premature aging with established aging-related cardiac dysfunction. Genome-wide total RNA-seq was performed and the expression patterns of protein-coding genes and non-coding RNAs were compared between hearts from naturally aging mice, mice with cardiac-specific deficiency of a component of the DNA repair machinery, mice with reduced mitochondrial antioxidant capacity and mice with reduced telomere length. Our results demonstrate that no dramatic changes are evident in the transcriptomes of naturally senescent murine hearts until two years of age, in contrast to the transcriptome of accelerated aged mice. Additionally, these mice displayed model-specific alterations of the expression levels of protein-coding and non-coding genes with hardly any overlap with age-related signatures. Our data demonstrate very limited similarities between the transcriptomes of all our murine aging models and question their reliability to study human cardiovascular senescence.

## Introduction

The perception of old age as a merciless and unavoidable perpetrator of the decay of the human body has been a common theme among ancient and modern artists. Oscar Wilde in his novel “*The Picture of Dorian Gray*” conveys the idea of the fading of youth coming with age as a despicable plague, as an illness consuming the body. Even the geneticist Richard Benedict Goldschmidt, yet with less harsh and colorful images, described aging as the unresolved, ultimate disease of the body; of Goldschmidt are the words “I have accumulated a wealth of knowledge in innumerable spheres and enjoyed it as an always ready instrument for exercising the mind and penetrating further and further. Best of all, mine has been a life of loving and being loved. What a tragedy that all this will disappear with the used-up body!”^1^.

From a biological perspective, aging still remains an unresolved dilemma: it is generally regarded as a complex process resulting from several causes, as epigenetic, proteotoxic and oxidative stresses, telomere shortening and unresolved DNA damages, all synergistically contributing to altering the functionality of cells, organs and ultimately of the entire organism^2–6^.

The most recent theories on aging hypothesize that the ultimate responsible for organismal senescence is the cumulative contribution of damages to DNA, proteins, lipids and organelles, produced either by intrinsic stress, such as reactive oxygen species (ROS) and reactive nitrogen species (RNS), or by extrinsic stress, such as UV light, irradiation and exposure to toxins, and together with other factors, namely genetic predisposition, epigenetic alterations, diet and physical activity^7^.

A big hurdle when studying the process of senescence is the choice of the model system to use. Longitudinal and cross-sectional studies on human aging are merely observational and not mechanistic in nature, thus making unavoidable the use of model organisms. For this purpose, rodents represent the unsurpassed gold-standard: mice and rats have a relative short lifespan (approximatively two years) which makes them easier to be used rather than long-lived animals^8^. Additionally, they offer the convenient advantage of genetic manipulation, which have resulted in the generation of the so-called “accelerated aging models”, carrying mutations that affect one or more of the pathways generally associated with physiological aging or reproducing human genetic afflictions of accelerated aging. The use of model organisms has however its downside: it is nearly impossible to tell whether an organism is representative of human aging or not since it is not known if the aging-related mechanisms operating across different species are conserved. This can cause pitfalls in the interpretation of the translational value of the findings in the current model organisms.

The constant increase in life expectancy together with low birth rates had caused a dramatic increase in the median age of the European population, making a better understanding of the aging process of critical relevance. In particular, studying the aging of the heart has become of great value, since among the elderly the prevalence of cardiovascular diseases is greatly enhanced. Indeed, with increasing age the prevalence of cardiovascular risk factors, such as metabolic syndromes sharply increases, promoting atherosclerosis and ischemic heart disease^9^. Preceding these structural modifications are changes in the “genomics” of the heart, as fluctuations in transcript abundance of protein-coding and non-coding genes and the re-expression of genes that are typically observed in the heart at the fetal stage^10,11^. A better understanding of the mechanisms driving the susceptibility to these pathologies is likely to be found by diving more in depth into the genomics of the heart. The efforts of the International Human Genome Sequencing Consortium (2004) have surprisingly revealed that the human genome encodes only 19,438 known protein-coding genes, accounting for about the 2% of total genomic sequence. Nonetheless, results from the Encyclopedia of DNA Elements (ENCODE) project reveals that at least 80% of this remaining non-coding fraction is functional, has a regulatory role and is transcribed into various classes of non-coding RNAs (ncRNAs)^12^.

Here, we investigated the genomic features of the aging murine myocardium. To this end, by total RNA-sequencing we charted the changes occurring in the transcriptome of murine heart tissue, taking into consideration both its protein-coding and non-coding fractions. To decipher the particulars of the aging process in the heart, we included cardiac tissue derived from a mouse model of natural aging and hearts from no less than three different mouse models of premature senescence, namely the heart-specific *Ercc1*-deficient model, characterized by an impaired DNA-repair machinery, the *Tert*-deficient model, lacking telomerase activity resulting in reduced telomere length, and the *Harlequin* (*Hq*) model that has increased vulnerability to oxidative stress. This last model is of particular interest for the variety of phenotypes that it develops, such as accelerated progression to heart failure in response to stress, sarcopenia and neurodegenerative changes^13^. Our results show a surprisingly high stability of the transcriptome of naturally aged mice throughout their lifespan and a remarkably small overlap of these expression patterns with those of the accelerated aging mouse models. Overall, we observed striking dissimilarities between prematurely aged mouse models and natural aging, which may raise doubts about the applicability of these models to the study of the genomics of human myocardial senescence.

## Methods

### Mouse models

The mouse models we used include a model of natural aging and three different models of accelerated aging, particularly mice with cardiac-specific deficiency of the *Excision repair cross-complementation group 1 (Ercc1)*, mice with reduced mitochondrial antioxidant capacity and *Telomerase Reverse Transcriptase* (*Tert)* deficient mice with reduced telomere length. Further details for each model are discussed below.

Male and female wild-type B6129F1 mice 12-, 52- and 104 weeks old were included as models of natural aging: the specific ages have been chosen to represent the young (12 weeks), adult (52 weeks) and old (104 weeks) phases of the murine lifespan. Wild-type B6129F1 mice show normal development and only in the oldest mice (104 weeks of age), mild signs of eccentric remodeling with fibrosis and slightly reduced contractility are evident^14^.

The first model of accelerated aging we included in the study is the one we address as the *Harlequin* (*Hq*) model: these are male mice on a B6CBACa-Aw-J/A (B6CBA) background between 17 and 20 weeks of age which present a proviral insertion in the first intron of *Apoptosis inducing factor mitochondria associated 1 (Aifm1)* in hemizygosity that reduces for the 80% the expression of this gene^15^. This gene encodes for the flavoprotein AIFM1: it is normally localized at the mitochondrial intermembrane space level where it plays a pro-survival role as free radical scavenger. These animals show clearly altered phenotypes, from neuronal loss, progressive retinal degeneration, ataxia to a reduced lifespan to less than 6 months, but no manifested cardiac abnormalities, apart from an increase in heart to body weight ratio^16^. When subjected to ischemia/reperfusion injuries, however, *Hq* mice show an increased cardiomyocyte apoptotic and necrotic death and accelerated progression towards maladaptive left ventricular remodeling if compared to wild-type animals^16^. This model is particularly relevant for aging since intrinsic oxidative stress increases with age in the heart, due to age-related impairment of transcriptional responses to oxidant stress, diminished expression of antioxidant defense enzymes such as catalase, glutathione peroxidase and MnSOD, increased myocardial expression of NADPH oxidases, culminating in age-dependent cardiac mitochondrial dysfunction^17–20^.

Another model of accelerated senescence we used for our study is the *Ercc1* heart-specific knock-out one: to establish this model, mice harboring a floxed allele of *Ercc1* in a 129P2/OlaHsd genetic background were crossbred with mice harboring Cre recombinase under control of the murine *Myh6* promoter in a C57BL/6 J background. For our purposes, we selected male animals of 16 weeks of age. As one of the main components of the nucleotide excision repair complex, Ercc1 is required for removing UV-induced DNA damages and inter-strand crosslinks^21^. Mice carrying the full-body knock-out of this protein show a very severe phenotype, with impaired growth and markedly reduced lifespan to a maximum of 8-10 weeks^22,23^; the heart-specific *Ercc1*-deficient mice we used in the present study show a milder phenotype than the one just described, a normal growth, a longer lifespan (maximum 23 weeks of age) and spontaneous cardiac alterations leading to dilated cardiomyopathy occurring over the first 16 weeks of life (unpublished observations Martine de Boer and Dirk Duncker).

The last progeroid model we used is the *Tert* knock-out mouse model: these animals are on a C57BL/6 J genetic background and were sacrificed at 16 weeks of age. In order to observe critical telomere shortening we used the third generation (G3) obtained after crossbreeding^24,25^. The TERT protein complex is responsible for *de novo* addition of telomeric repeats at the ends of chromosomes; in mice and humans, telomerase is normally silenced after birth. As a consequence, a slow, progressive telomere shortening is observed throughout the lifespan that subsequently triggers cellular senescence and apoptosis-related pathways^26–28^. *Tert*-deficient mice interbred for four generations (G4) to lower telomere reserves manifest ventricular dilation, ventricular wall thinning and cardiac dysfunction^29^. Mice used in the current study are of the third generation (G3), which have a less pronounced and milder cardiac phenotype as described for G4 Tert-deficient mice (unpublished observations Christian Bär).

All protocols were performed according to institutional guidelines and approved by local Animal Care and Use Committees. Specifically, these authorities included the institutional animal care and user committee of the Lower Saxony State Office for Consumer Protection and Food Safety in Germany (license number 2017/143), the Institutional animal care and use committee at Université Paris Descartes (Plateforme d’hébergement et d'élevage des Saints-Pères, license number C 75-06-07) and the Dutch Central Committee of Animal Use (CCD) and Institutional animal care and user committees at Erasmus MC and Maastricht University in accordance with the respective National Authorities in Germany, France and the Netherlands and guidelines based on European Union Directive 2010/63/EU. All mice were housed on a 12 hr light:12 hr light:dark cycle in a temperature-controlled environment with *ad libitum* access to water and chow with a quarterly animal health monitoring system that complies with FELASA guidelines and recommendations. Randomization of subjects to experimental groups was based on a single sequence of random assignments. Animal caretakers blinded investigators to animal group allocation before NGS sequencing and/or when assessing the outcome.

### RNA isolation

Total RNA was extracted from myocardial tissue of mice euthanized at the timepoints reported above using the Direct-zol™ RNA MiniPrep method (Thermofisher) following the manufacturer’s protocol.

### RNA sequencing

All samples were prepared simultaneously during all steps of this analysis in order to exclude introduction of technical variability. Quality control of total RNA was performed using the RNA 6000 Pico Kit (Agilent Bioanalyzer) yielding in RIN values of 6.3 and higher. Removal of rRNA was carried out by use of the NEBNext rRNA Depletion Kit Human/Mouse/Rat (NEB) followed by strand-specific cDNA NGS library preparation (NEBNext Ultra II Directional RNA Library Prep Kit for Illumina, NEB). The size of the resulting library was controlled by use of a D1000 ScreenTape (Agilent 2200 TapeStation) and quantified using the NEBNext Library Quant Kit for Illumina (NEB). Equimolar pooled libraries were sequenced in a paired end mode (75 cycles) on the NextSeq. 500 System (Illumina) using v2 chemistry yielding in an average QScore distribution of 84%>= Q30 score. After quality control with FastQC v0.11.2^30^ raw sequencing data was read trimmed using trimmomatic v0.36^31^ and aligned to the mouse reference genome GRCm38 (ensemble release 90) with hisat2 v2.1.0^32^. Overall alignment rate was observed between 70–84% for all samples. Gene assembly was done using stringtie v1.3.4d^33^ with set ‘-e’ parameter to produce read count matrices for reference genes and transcripts. The complete RNA-seq data sets from this study were deposited at the Gene Expression Omnibus (GEO) with reference GSE124087 in MINSEQ-compliant data submission format under restricted release date and will become publicly available pending acceptance of the current manuscript.

### cDNA synthesis

Reverse transcription of the total RNA was performed using M-MLV (Promega) with oligo(dT) primers (Invitrogen) and random hexamers (IDT) following the manufacturer’s protocol.

### Real-Time PCR

Real-time PCR was performed on a Bio-Rad iCycler (Bio-Rad) using SYBR Green. Transcript quantities were compared using the relative Ct method, where the amount of target is normalized to the amount of endogenous control (L7) and expressed relative to the control sample (12 week-old naturally aged animals) as 2^−ΔΔCt^. The sets of primers used are reported in Supplementary Table 1.

### Differential expression analysis and statistical analysis

After evaluation of principal component analysis, two samples showed outlier characteristics. Because this could not have been explained by technical differences, all samples were used for further analysis for better transparency. Differential expression analysis was performed using the DESeq2 R-package version 1.20.0^34^ applied on the gene count matrix obtained from stringtie. The count matrix was normalized for library size and lowly expressed genes (minimum rowsum <10) have been removed from the dataset. Gene biotypes were annotated using the R annotation package org.Mm.eg.db version 3.6.0. All analyses have been applied to the full data set as well as to data subsets containing protein-coding genes or lncRNA genes only. Genes were considered as significantly differentially expressed if the corresponding absolute fold change was greater than 1.5 and the differential expression P-value was less than 0.05 after adjustment for false discovery rate according to the method of Benjamini and Hochberg. Expressed genes were analyzed in an over-representation analysis for enrichment of Reactome pathways and gene ontology (GO) terms using the top 100 differentially expressed genes, as it’s implemented in the clusterProfiler package version 3.8.1^35^. The top 5 enriched GO terms of each analysis have been visualized with their corresponding genes as gene-concept networks using the cnetplot function of clusterProfiler.

### The results of the Real-time PCR analysis are presented as mean ± SEM

Statistical analyses were performed using Prism software (GraphPad Software), and consisted of ANOVA followed by Bonferroni’s multiple-comparison test when group differences were detected at the 5% significance level or Student’s t test when comparing two experimental groups. Differences were considered significant when p < 0.05.

### Principal component analysis and hierarchical clustering

Further downstream analysis was performed on variance stability transformed count data. Principal Component Analysis (PCA) was performed to broadly understand the gene expression relationship between models and visualize sample clustering for the most variably expressed genes. Loading scores and explained variance for the first two principal components were calculated and PCA plots were generated using the plotPCA function implemented in DESeq2. Heatmaps were produced using the significant genes of each group comparison and hierarchical clustering. Heatmap plots was generated using the heatmap.2 function in the R package gplots. Row Z scores were calculated for better gene expression comparison between samples, meaning that for each gene, the average intensity across all samples is set to zero and standard deviation is set to 1. A color code of + −1 means + − 1 standard deviation from the mean. The dendrogram was constructed based on the average distant algorithm implemented in heatmap.2.

## Results

### The transcriptome remains relatively stable in the naturally aged myocardium

**Figure 1:**
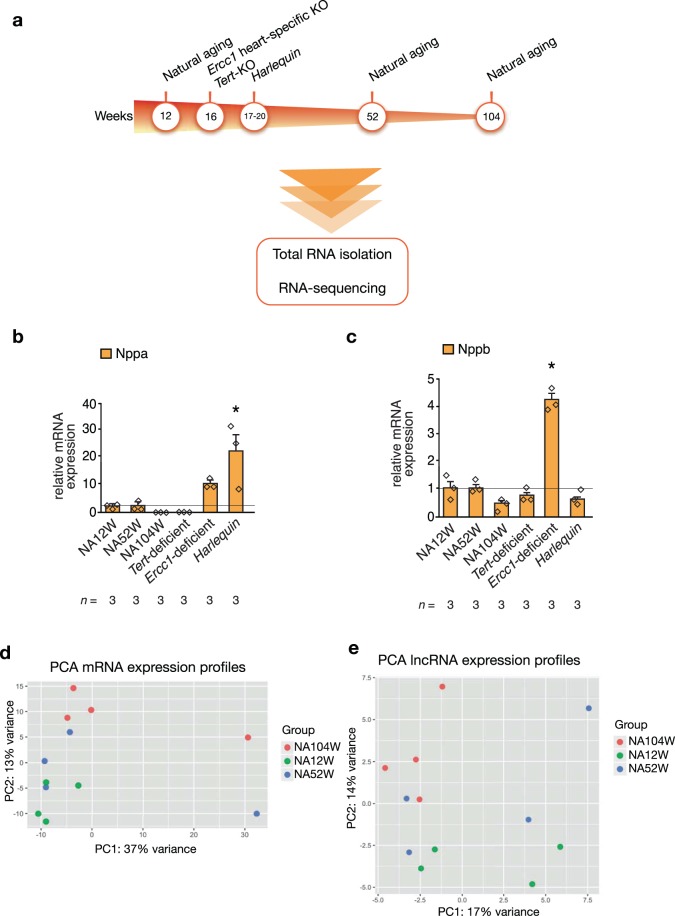
Experimental design and PCA plots of mRNAs and lncRNAs in the naturally aging model. **(a)** Experimental timeline followed for the collection of the heart tissues used for the RNA-sequencing. Real-time PCR expression analysis of the cardiomyocytes stress genes **(b)** *Nppa* and **(c)** *Nppb* in hearts from mice modeling natural and premature senescence, *n* refers to number of hearts. PCA plots of the **(d)** mRNAs **(e)** lncRNAs data representing the expression profiles trends of the hearts of naturally aged mice of 12, 52 and 104 weeks of age. The plots have been generated using the first two principal components, which together account for the **(d)** 50% and **(e)** 31% of the variance between samples, *n* = 4 hearts for each mouse model. NA12W, natural aging 12 week-old; NA52W, natural aging 52 week-old; NA104W, natural aging 104 week-old; *P < 0.05 vs wild-type 12 week-old mice.

To study the effects of natural aging on the genomic features of the heart, we performed total RNA sequencing on hearts from naturally aged mice of 12, 52 and 104 weeks of age and of three mouse models of accelerated aging: *Ercc1* heart specific knock-out mice, *Tert* knock-out mice and mice harboring the *Hq* mutation characterized by haploinsufficiency of *Aifm1* (Fig. 1a). A technical summary of the RNA-seq is reported in Table 1: for each group sample, an average of 100 million paired reads were generated, accounting for an alignment rate varying from a minimum of 74,4% for the *Ercc1*-deficient mouse model to a maximum of 81,7% for the *Tert*-deficient and the young, naturally aged models. Of the paired reads, a percentage in-between 21,8% and 29,7% did not map concordantly to the genome, i.e. either the read pair maps discordantly or just one read of the pair maps to the genome, the 63,5-70,8% of read pairs aligned concordantly only once and the 6,8-8,4% aligned concordantly more than once. In each group, ~32 million of reads were mapped to protein-coding gene loci, 14 to 20 million were mapped to lncRNA genes and 34–44 million were mapped to other regions of the genome.

**Table 1:** Technical summary of the RNA-seq mapping results.

	Ercc1-deficient	Harlequin	Tert-deficient	NA12w	NA52w	NA104w
Single reads	58,573,980	57,188,219	58,083,642	56,299,051	55,973,585	59,310,196
Overall alignment rate	74,40%	79,20%	81,70%	81,70%	80,50%	80,60%
Pairs aligned concordantly 0 times	29,70%	24,80%	21,80%	21,90%	23,10%	22,90%
Pairs aligned concordantly exactly 1 time	63,50%	66,80%	70,80%	70,60%	69,50%	69,40%
Pairs aligned concordantly > 1 times	6,80%	8,40%	7,40%	7,50%	7,40%	7,70%
Reads mapping to protein-coding genes	3,24E + 07	3,20E + 07	3,45E + 07	3,18E + 07	3,13E + 07	3,43E + 07
LncRNA genes	2,04E + 06	1,36E + 06	1,54E + 06	1,47E + 06	1,46E + 06	1,48E + 06
Others	3,35E + 07	4,40E + 07	3,50E + 07	3,75E + 07	3,61E + 07	3,89E + 07

The degree of cardiac dysfunction described for the mouse models of aging used in this study is remarkably varied: naturally aged and *Tert*-deficient mice share a relatively mild cardiac phenotype (mild eccentric remodeling, slight reduction in contractile function), *Hq* mice have slightly bigger hearts and a worsening in the response to ischemia/reperfusion injuries and *Ercc1*-deficient animals develop dilated cardiomyopathy. To assess the extent of this heterogeneity we chose to analyse the expression levels of the cardiomyocyte fetal genes *Nppa* and *Nppb*. In physiological conditions, postnatal expression of *Nppa* and *Nppb* is strongly downregulated; however, it gets drastically increased during adverse structural remodeling. The results of the Real-time PCR analysis of *Nppa* and *Nppb* are reported in (Fig. 1b,c): a significant up-regulation of *Nppa* was found only in the *Hq* model, although a similar trend is observed also for the hearts from *Ercc1*-deficient animals. As for *Nppb*, its expression is significantly increased only in the *Ercc1*-deficient model. However, neither *Nppa* nor *Nppb* are differentially expressed in the *Tert*-deficient and in the natural aging models. These results confirm the disparities in the cardiac phenotypes manifested by these models of senescence, with *Ercc1*-deficient and *Harlequin* mice having the most disease-like ones. Additionally, these data are suggestive of a remarkably healthy-like transcriptional profile of 104 week-old naturally aged hearts, that we further investigated by comparing the mRNAs, lncRNAs and miRNAs differentially expressed between the three time-points of natural aging.

Starting from the protein-coding genes profiles, there was no clear separation between the transcriptomes of the murine hearts of different ages when looking at the first component of our principal component analysis (PCA); in fact, we could only detect mild intra-group variance accounting for expected small differences between biological replicates (Fig. 1d). Only when considering the second component, which describes just 13% of the variance between the samples, a separate clustering between the three time-points of aging became slightly more evident. Similarly, no detectable clustering of the three timepoints of natural aging could be detected when performing the PCA on the lncRNA data (Fig. 1e). Our sequencing analysis provided us also with information on the miRNAs expressed in the hearts of naturally aging mice; however, when comparing the different time-points, we did not detect any differentially expressed miRNAs. Conclusively, the expression profiles of all the RNA biotypes we detected with our sequencing approach remained remarkably stable throughout the life span of naturally aged mice.

### Quantitative differences in the transcriptomes between naturally- and accelerated aged hearts

**Figure 2:**
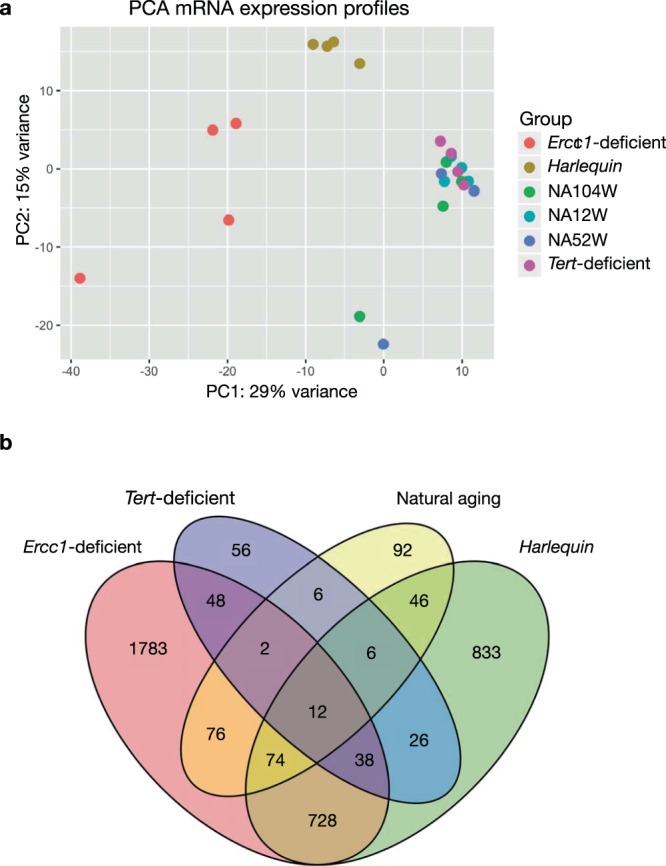
MRNA expression profiles in the natural and premature aging models. **(a)** PCA plot summarizing the trends in the mRNA expression profiles of the hearts of the different models of senescence. The plot has been generated using the first two principal components, which together account for the 44% of the variance between samples, *n* = 4 hearts for each mouse model. **(b)** VENN diagram showing the number of significantly differentially expressed mRNAs in each aging model in comparison to the wild-type youngest timepoint. NA12W, natural aging 12 week-old; NA52W, natural aging 52 week-old; NA104W, natural aging 104 week-old.

Next, we characterized the transcriptomic profiles of three mouse models of accelerated aging: *Ercc1* heart specific knock-out mice, *Tert* knock-out mice and mice harboring the *Hq* mutation characterized by haploinsufficiency of *Aifm1* (Fig. 1a). Specifically, our interest was to verify the similarity of these accelerated aged models to their naturally aged counterpart. Our results demonstrate that the profiles of the accelerated aging models *Ercc1*-deficient and *Hq* markedly differ from the *Tert*-deficient model and naturally aged animals. Compared to the variation detected between the different models of accelerated aging, the transcriptome profiles of naturally aged mice of 12, 52 and 104 weeks of age cluster very close together, indicating that the differences in protein-coding gene expression profiles within these groups is considerably smaller than those between any of the models of premature senescence except for the *Tert*-deficient model (Fig. 2a). These observations suggest a minimal similarity between the transcriptomes of naturally- and accelerated aging models, an unexpected finding considering that the prematurely aged models, per definition, were expected to manifest, at least in part, similar changes during physiological senescence but at an accelerated pace. Indeed, the transcriptomes of the *Ercc1*-deficient model and the *Hq* model, when compared to age-matched, wild-type mice, involved a much larger number of differentially expressed genes than those altered in the naturally aged mice (Fig. 2b**)**. Even though a slight overlap in changes in expression levels of protein-coding transcripts could be detected between natural and accelerated aging, the modifications in the transcriptomes of the models of premature senescence were strikingly bigger and model-specific, suggesting that the biological processes involved in the pathophysiology of accelerated aging are most likely more numerous and more robustly altered. Among the premature senescence models, the *Tert*-deficient mouse model showed a transcriptome profile that was most similar to natural aging.

**Figure 3:**
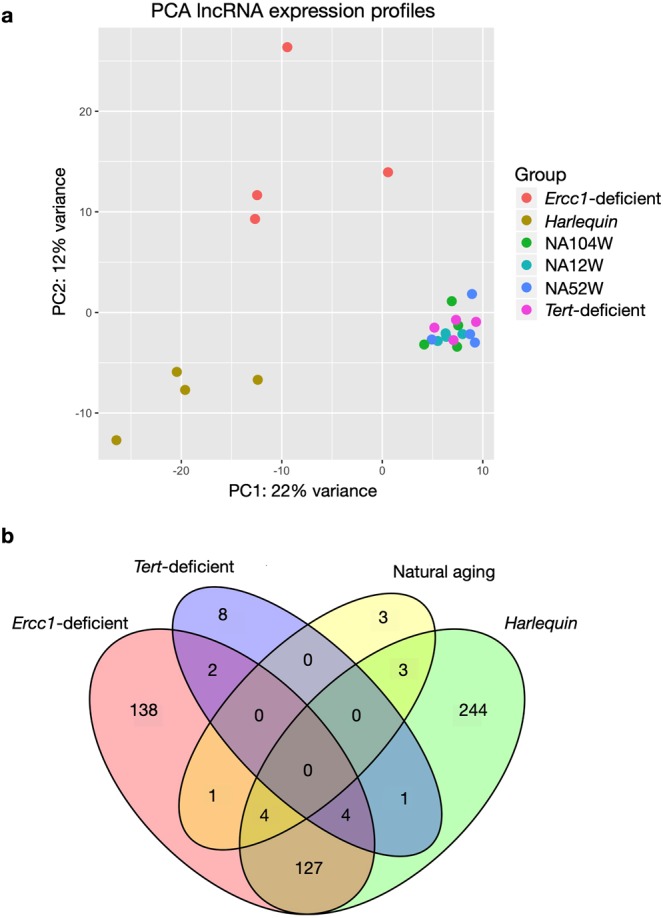
LncRNA expression profiles in the natural and premature aging models. **(a)** PCA plot summarizing the lncRNA expression profiles of the hearts of the different models of senescence. The plot has been generated using the first two principal components, which together account for the 34% of the variance between samples, *n* = 4 hearts for each mouse model. **(b)** VENN diagram showing the number of significantly differentially expressed lncRNAs in each aging model resulting from the comparison to the wild-type youngest timepoint. NA12W, natural aging 12 week-old; NA52W, natural aging 52 week-old; NA104W, natural aging 104 week-old.

The same observations hold true when analyzing the expression profiles of lncRNAs (Fig. 3). LncRNA transcriptomes from the *Ercc1*-deficient and *Hq* models were more different compared to the natural aging and *Tert*-deficient models, whilst the latter tended to cluster close together (Fig. 3a). Even quantitatively, the alterations in lncRNAs in the *Ercc1*-deficient and *Hq* models involved a considerable larger number of transcripts than those observed for the *Tert*-deficient model or in naturally senescent animals. Moreover, the majority of the differentially expressed lncRNAs were altered in a model-specific manner, supporting the contention that in each model distinct pathophysiological processes are operative (Fig. 3b).

Taken together, our findings point to a stark disparity between the transcriptomes of the three different accelerated aging models, which, additionally showed marked dissimilarities when compared to the transcriptome of naturally aging animals.

### Dramatic changes in the transcriptome of *Harlequin* mouse hearts

**Figure 4:**
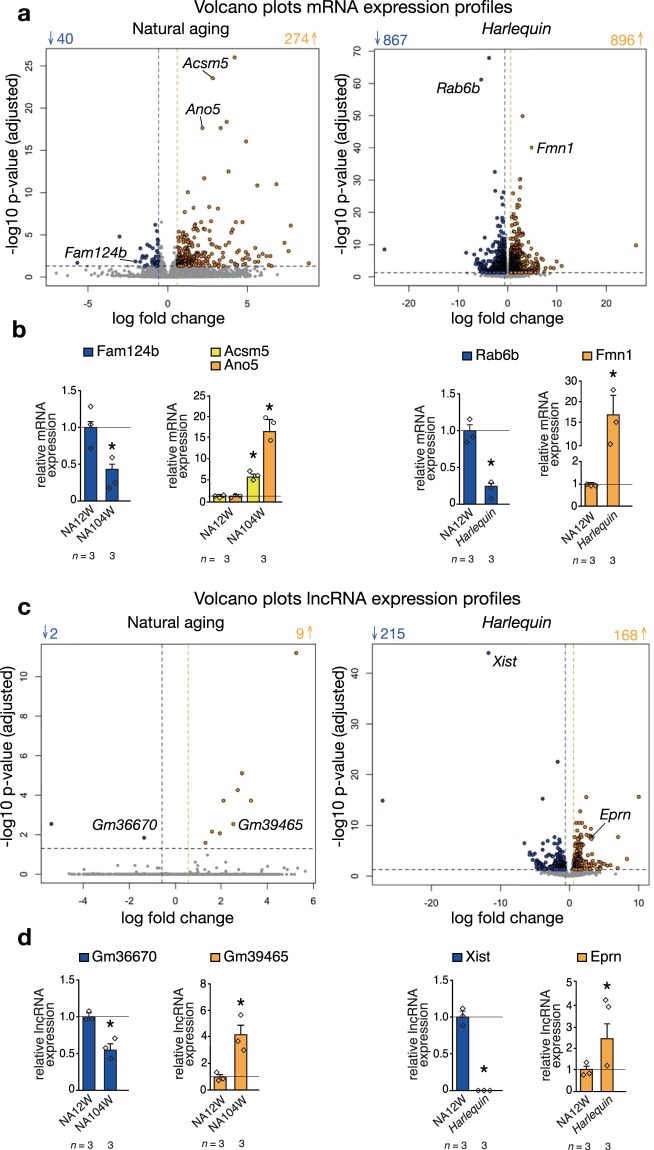
Representation of the differences in the expression profiles between selected group comparisons. Volcano plots reporting the differentially expressed **(a)** mRNAs and **(c)** lncRNAs in the hearts of naturally aged and *Harlequin* mice compared to wild-type 12 week-old animals. In blue and yellow are the down- and up-regulated genes respectively. **(b-d)** Real-time PCR analysis of selected targets validates the RNA-sequencing results. *P < 0.05 vs wild-type 12 week-old mice; *n* refers to *n*umber of hearts.

Among the models of premature senescence, the *Harlequin* model is particularly interesting to study the aging process in general, given the variety and severity of the phenotype developed in this model, and of the heart in particular, given the crucial role that intrinsic oxidative stress plays in the genesis of senescence. As previously stated, we observed that the hearts of naturally aged mice maintain a relatively stable transcriptome over time; indeed, only 314 protein-coding genes were differentially expressed between 12 and 104 week-old hearts, of which 274 were downregulated and 40 upregulated (Fig. 4a). These numbers are dwarfed when compared to the number of genes altered in the *Hq* model: 1763 in total, of which 867 genes were downregulated and 896 were upregulated (Fig. 4a).

This dichotomy became even more evident when differentially expressed lncRNAs were considered (Fig. 4c). The numbers of lncRNAs significantly altered in expression in the young versus old naturally aged hearts were extremely low (11 in total, of which 9 downregulated and 2 upregulated), while a total of 383 lncRNAs were differentially expressed in *Hq* hearts, of which 215 were downregulated and 168 were increased in expression.

We validated the RNA sequencing data by performing Real-time PCR analysis of targets selected among the most differentially regulated protein-coding transcripts and lncRNAs in both group comparisons (Fig. 4b,d). Our sequencing results are strongly supported by Real-time PCR data since we confirmed the same trends of expression in a statistically significant way.

Taken together, these results underline that the transcriptome during natural aging remains stable, which contrasts to the dramatic alterations found in the expression profile of hearts from *Hq* mice.

### The transcriptomes of accelerated aging models diverge from natural aging

**Figure 5:**
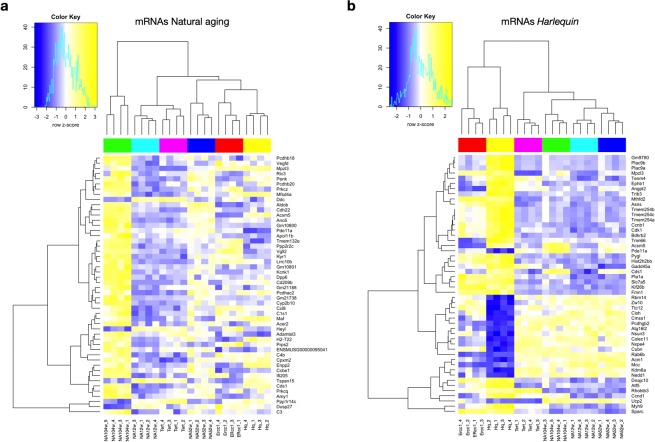
Heatmaps of differentially expressed mRNAs. Heatmaps of the 50 most differentially regulated mRNAs resulting from the comparison between **(a)** young and old naturally aged hearts and between **(b)** the *Harlequin* model with its age-matched counterpart; the normalized z-scores of the count data are shown for all the aging models used, *n* = 4 hearts for each mouse model.

**Figure 6:**
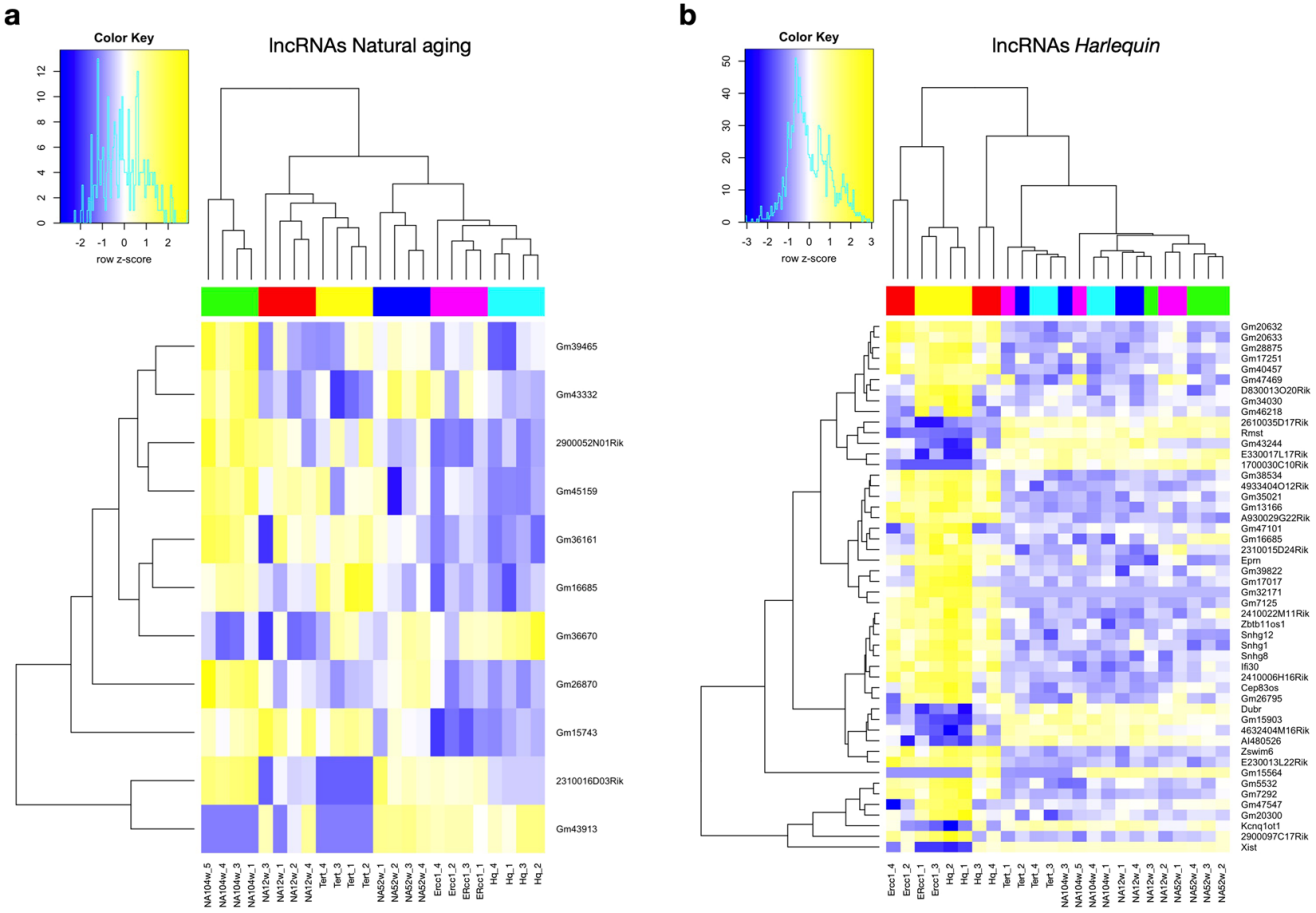
Heatmaps of differentially expressed lncRNAs. Heatmaps of the 50 most differentially regulated lncRNAs resulting from the comparison between **(a)** young and old naturally aged hearts and between **(b)** the *Harlequin* model with its age-matched counterpart; the normalized z-scores of the count data are shown for all the aging models used, *n* = 4 hearts for each mouse model.

Next, the grade of transcriptome overlap between the distinct models of senescence was analyzed. In theory, as models of the same process, their expression profiles were expected to be largely overlapping. Surprisingly, our results depict radical differences between the various models. Figure 5a depicts the normalized z-scores of the count data in all premature aging models of the 50 most differentially regulated protein-coding genes found in the comparison between young and old naturally aged hearts. As expected, the expression pattern of 52 weeks old hearts was an intermediate version of the young and old hearts. More unexpectedly, the data further shows that the models of premature aging are either clustering with the young (*Tert*-deficient) or adult (*Ercc1*-deficient, *Hq*) natural aging timepoints rather than with old one. The same observations hold true when analysing the fold changes of the lncRNAs in all aging models (Fig. 6a). 52 week-old hearts manifest an intermediate pattern compared to young (12 weeks) and old (104 weeks) hearts and the transcriptomes of the prematurely aged models cluster closer with the young and intermediate aged hearts rather than with the eldest time point.

This discordance between naturally- and accelerated aged cardiac transcriptomes became even more evident from the heatmaps representing the 50 most altered protein-coding genes (Fig. 5b) and lncRNAs (Fig. 6b) resulting from the primary comparison between *Hq* and age-matched naturally aged hearts. The same heatmaps also report the level of expression of the same transcripts in the other aging models. For both protein-coding genes and lncRNAs, the transcriptome of the *Hq* model stands clearly apart from all time-points of natural aging and also differs greatly from that of *Tert*-deficient mice, while being relatively similar to *Errc1*-deficient animals (Fig. 5b, Fig. 6b). Interestingly, the majority of most dysregulated genes in the hearts of *Hq* mice are not or only mildly altered in the other models, confirming that the genomic alterations observed are specifically occurring in response to the *Hq* mutation. As an additional layer of information, we gathered in (Supplementary Tables 2,3) the list of protein-coding genes within 1Mbp from the loci of the lncRNAs reported in (Fig. 6); this can provide meaningful information for *cis*-acting lncRNAs.

Taken together, these data demonstrate an unexpectedly limited overlap of the transcriptomes of accelerated aged models between each other and reinforce the contrast with the senescent, naturally aged animals.

### Functional enrichment analysis of the aging models confirms their biological dichotomy

**Figure 7:**
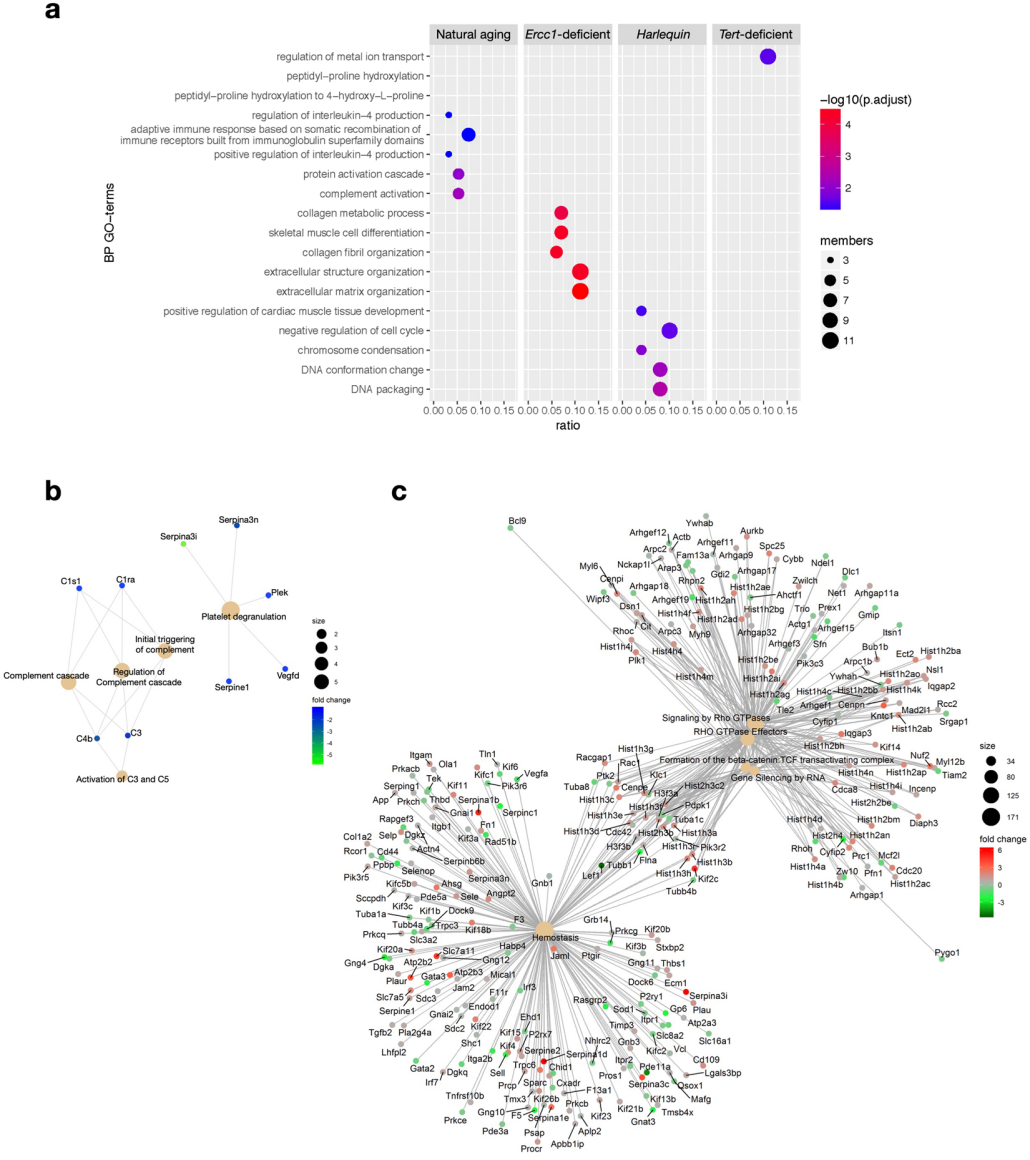
Functional enrichment analyses of aging models. **(a)** GO-terms enrichment analysis using the differentially expressed mRNAs in each aging model after the comparison with wild-type 12 week-old mice. Gene-Concept networks visualizing the individual protein-coding genes associated to the GO-terms found enriched for **(b)** naturally-aged and **(c)** *Harlequin* mice.

Finally, we explored the biological processes affected by the alterations in gene expression observed in each of our aging models. Accordingly, we first performed a GO-terms enrichment analysis considering the top 100 differentially expressed protein-coding genes between the accelerated aging models and their young reference wild-type counterpart. Separately, we also considered a GO-terms enrichment of genes altered between young and old naturally aged animals. On the basis of these gene expression patterns, we performed the analysis for the enriched GO terms belonging to the Biological Process (BP) category. The dot-blot visualizes the results for the top 5 enriched GO terms for every group comparison and the most altered biological processes in each model (Fig. 7a). Remarkably, the biological processes altered in each model were non-overlapping. Specifically, of the 18 GO terms included in the graph, those enriched in naturally aged animals related to the immune reactivity and complement activation, an observation in agreement with the concept of immunosenescence, the decline of innate and adaptive immune responses occurring with age, a phenomenon extensively described in literature^36,37^. The GO terms enriched for the *Ercc1*-deficient model mostly specify for extracellular matrix organization and collagen fibril assembling, reflecting the fibrotic tissue deposition accompanying dilated cardiomyopathy, which develops in the hearts of *Ercc1*-deficient animals. For *Hq*, the GO terms most enriched are those referring to chromosome condensation and DNA packaging, possibly reflecting the damaging effect of reactive oxygen species on DNA integrity^38^.

Next, we created a cnet plot to visualize the individual genes underlining the biological phenomena found altered in naturally aged hearts: *C1s1*, *C1ra*, *C4b* and *C3*, responsible of the regulation of the complement cascade, were found differentially expressed, as well as limited numbers of *Serpin* genes (e.g. *Serpina3i*, *Serpina3n*, *Serpine1*), implicating fibrinolysis and platelet degranulation in the natural aging of the myocardium (Fig. 7b). When juxtaposing these results with the cnet plot generated for the *Hq* model, the difference in the number of genes implicated was striking (Fig. 7c). Here, numerous genes formed clustered networks revolving around Rho GTPase activity (e.g. *Cdc8a*, *Iqgap3*), chromatin remodeling (e.g. *Hist1h2a1*, *Hist1h4m*, *Hist1h3g*) and nuclear signaling cascades (e.g. *Lef1*, *Ywhah*). Likewise, matrix and transmembrane constituents (*Tuba1a*, *Fn1*, *Itga2b*), several protein kinase C isoforms (*Prkcg*, *Prkacb*, *Prkch*), cardiac-specific cytoskeletal components (*Tln1*, *Actn4*) as well as intracellular signaling mediators (*Pde5a*, *Pde11a*), also known for their effect on platelet function, were found as central nodes of the *Hq* network.

The GO terms higlighted in this analysis can be efficiently contextualized within the unique phenotypes of each aging model, suggesting that the biological processes that were altered in each model are not driving them more closely to the natural aging condition, but rather generate an exclusively pathological state strictly correlating to the mutation that has been introduced by the generation of the model itself.

## Discussion

Here, we provided a description of the changes in the transcriptome of the murine heart during aging. To this end, we analysed by RNA-seq the cardiac expression profiles of protein-coding and non-coding genes in naturally aging mice of 12, 52 and 104 weeks of age and in three different mouse models of accelerated aging, each characterized by the alteration of one of the pathways regarded in literature as crucial contributors to senescence. Specifically, we included mice with cardiac-specific deficiency of *Ercc1*, encoding a component of the DNA repair machinery, *Hq* mice with reduced mitochondrial antioxidant capacity and *Tert*-deficient mice with reduced telomere length.

A first, striking result emerging from our analysis is that the transcriptomes of the models of premature senescence dramatically differ from each other and from the reference wild-type counterpart. Although arising from alterations of different biological processes, the accelerated aging models should display a similar “aging behavior”, since the pathways that have been mutated to generate these models are all extensively reported in literature as contributing to senescence. Accordingly, even if genetically different, these models should ultimately reproduce the same phenomena and manifest in a shorter period of time what results from a lifetime of intrinsic and extrinsic insults in a naturally aged animal. In stark contrast, our results depict a different scenario: from the perspective of their transcriptomes, not only do the accelerated aging models largely differ from the eldest time point of natural aging but they also show specific features that set each model apart from the rest. One possible explanation is that the extent of the alteration caused when genetically manipulating these animals is so dramatic to produce a pathological outcome rather than a physiological phenotype resembling the one observed at old age. Ultimately, this leads to the conclusion that each of these models is manifesting the pathology caused by the impairment of a certain cellular pathway and not an accelerated aged phenotype produced by aggravating one of the proposed molecular causes of senescence.

A second and arguably even more controversial aspect highlighted by our results is that naturally aging mice do not display large changes in their cardiac gene expression profiles over time. As widely reported in literature, a commonly used animal model to study aging is the mouse; accordingly, we tackled physiological senescence by considering mice at three stadia, 12, 52 and 104 weeks of age, so to have a comprehensive overview of the murine transcriptome over its lifespan. In contrast to our expectations, we observed a surprisingly stable expression of protein-coding genes and lncRNAs and, when considering miRNAs, we were not able to detect any differentially expressed transcript over time. We tend to exclude that the reason for these observations is the choice of a too premature age for the eldest timepoint of our study, as 24 months is supported by previous reports as a representative old age in mice in the context of cardiac senescence^39,40^. Our findings point instead to the conclusion that the phenomenon of physiological aging in the murine heart fails to recapitulate myocardial aging as observed in humans. Supporting this hypothesis is the fact that laboratory mice do not display a higher incidence of cardiac diseases nor typical age-related comorbidities as their natural cause of death but rather succumb from neoplasia with a very rare incidence of cardiovascular abnormalities^41,42^. Conversely, in humans, as reported by the World Health Organization (WHO), of the 56.9 millions deaths worldwide in 2016, 15.2 millions where caused by ischemic heart disease and stroke. The translational value of laboratory mice to model advanced age-related human heart diseases is, therefore, debatable.

There are three main limitations to the current study. First, the degree of cardiac dysfunction described for the aging mouse models in this study is remarkably variable. The naturally aged and the Tert-deficient mouse models share a relatively mild cardiac phenotype (mild eccentric remodeling, slight reduction in contractile function), Harlequin-deficient mice show spontaneous hypertrophic remodeling, while the Ercc1-deficient model displays a severe dilated cardiomyopathy at the age analyzed^14,16,29^. These models were previously characterized in terms of hemodynamic assessment and myocardial histopathology and these analyses were not repeated in this study for logistical reasons. Nevertheless, despite the lack of a direct coupling of our RNA-seq data with pathophysiological characterization, we could confirm the disparities in the cardiac phenotypes manifested by these models of senescence by assessing transcript abundance of the genes *Nppa* and *Nppb*, robust markers of adverse structural remodeling.

Secondly, in this study we relied on bulk RNA-sequencing that integrates transcriptomic information across thousands of cells and results in averaged gene expression levels. Although an average expression level for each gene in the cell population can be sufficient in many applications, such as to determine disease biomarkers or to compare the overall transcriptomic differences between disease states, as performed in the current study, bulk RNA-seq analysis lacks the detail of cell-specific functionality within a specific model. The adult murine myocardium is composed of approximately 30% myocytes, 40% endothelial cells, 15% hematopoietic-derived cells and 15% fibroblasts. Bulk RNA-seq lacks of cellular resolution, which might account for possible differences with other studies using purified myocardial cells^43^. For a future in-depth analysis, the diverse cell types of each model in this study could be sequenced, but this endeavour falls beyond the scope of the current study.

A third potential limitation relates to the genetic background of the mouse strains used. In the most optimal scenario, all models would have been of the exact same, pure inbred background. For practical reasons, the hybrid background B6129F1 for the naturally aging mouse strain was deliberately chosen as only this hybrid strain shows signs of cardiac dysfunction and interstitial fibrosis at advanced ages (e.g. 24 months) as opposed to the age-resistant pure inbred C57BL6/J strain^14^. To correct for slightly altered differences in the transcriptome due to background differences, strain- and organ-specific gene expression profiles would need to be generated and later integrated into RNA-seq pipelines to filter and normalize the sequencing results on the basis of these patterns. This kind of analysis has been repoted for a select number of inbred strains, but not for the hybrid strains used in this study^44^. Studies aimed at unraveling strain-specific gene expression profiles were indeed able to find differences at the genomic (SNPs), transcriptomic (mRNA) and proteomic levels but failed to trace them on all levels simultaneously^45,46^. For example, Parks and coworkers showed 160 differential strain specific expressed proteins when comparing C57BL/6 J and DBA/2 J inbred mouse strains. However, of those only 26% showed significant strain differences at the mRNA level whilst the 76% demonstrated the same strain effect direction^45^. These results lead us to the assumption that, although different strain background could have an influence when being compared, their influence should be irrelevant when comparing model-specific effects as performed in this study.

Based on our findings, we speculate that a more appropriate murine model of human cardiac aging would include vascular diseases (e.g. atherosclerosis), possibly in combination with high blood pressure and metabolic disorders. One strategy for the generation of such a model would be to introduce the comorbidities that are frequently observed among elderlies but absent in laboratory mice. The incidence of cardiovascular risk factors, such as high blood pressure and metabolic syndrome, hyperlipoproteinemia and type 2 diabetes, sharply increase with age among humans; mice, on the contrary, rarely spontaneously manifest these comorbidities. This difference could account for the disparity between mouse and human cardiac aging, although this contention would require a side-by-side confirmation of a similar transcriptomic analysis of human hearts over their lifespan. Conclusively, our study proposes controversial points of discussion on the reliability of the most commonly used murine aging models to study human cardiac senescence.

## Supplementary information

Supplementary information
