# Supplementary material for: Dichotomy between the transcriptomic landscape of naturally versus accelerated aged murine hearts
Source: Sci Rep. 2020 May 18;10:8136. doi: 10.1038/s41598-020-65115-9 (PMC7235007; doi:10.1038/s41598-020-65115-9)
Supplement: Supplementary file 1 — Supplementary information [file 41598_2020_65115_MOESM1_ESM.pdf]

Supplementary information to:

Federica De Majo, Jana-Charlotte Hegenbarth, Frank Rühle, Christian Bär, Thomas Thum, Martine de Boer, Dirk J. Duncker, Blanche Schroen, Anne-Sophie Armand, Monika Stoll, Leon J. De Windt. *Dichotomy between the transcriptomic landscape of naturally versus accelerated aged murine hearts.*

Supplementary Table 1*: Primers Real time PCR*

Supplementary Table 2*: Differentially expressed lncRNAs and their neighbouring genes in naturally aged 104 week-old mice*

Supplementary Table 3*: Top 50 differentially expressed lncRNAs and neighbouring genes in Harlequin mice*

Supplementary Table 1. Primers Real-time PCR

Fam124b FWD	CTTGAACGGCTGCTTGAGAG
Fam124b RV	CTTTGAACCTCCGACTCCTG
Acsm5 FWD	CACACACAGCCACCACCT
Acsm5 RV	TCCACACATCCAACACATCA
Ano5 FWD	ATTCTCCCTCCTCCTCCTGT
Ano5 RV	ACCCACATCCTCCCTTTAGC
Rab6b FWD	CTGCTGGGCAAGAGAGGTT
Rab6b RV	GTCTGCTGGAAGGAGTTGAGA
Fmn1 FWD	AGCAAAGCAACCAGAAGCAG
Fmn1 RV	GTGGAGGAATGAGAGGTGGA
Gm36670 FWD	CTGGCGACTTCTGCTGCTA
Gm36670 RV	ATTTCCTCCCGTGCTTATCA
Gm39465 FWD	GCCCTGAGTTTGGATTGC
Gm39465 RV	CCTGGAGTTCGCTGGTTC
Xist FWD	TCAGGATTCAAGTGGCTCTG
Xist RV	TCTTCTTTGGGTTGTCAGCA
Eprn FWD	TAGTCCAGAGGATGGGCAAC
Eprn RV	CACCAGAGGTAGTGGGAAGG
Nppa FWD	TCTTCCTCGTCTTGGCCTTT
Nppa RV	CCAGGTGGTCTAGCAGGTTC
Nppb FWD	TGGGAGGTCACTCCTATCCT
Nppb RV	GGCCATTTCCTCCGACTTT
L7 FWD	GAAGCTCATCTATGAGAAGGC
L7 RV	AAGACGAAGGAGCTGCAGAAC

Supplementary Table 2. Differentially expressed lncRNAs and their neighbouring genes in naturally aged 104 week old mice.

MGI Symbol	***Ensembl ID***	***Locus***	***Genes (1,000,000bp)***
Gm45159	ENSMUSG00000109125	Chr7:90,887,072-90,940,052	Dlg2,Syt12,Picalm,eed, Hikeshi,Ccdc89, Crebzf, Tmem126a, Tmem126b, Ccdc83
Gm43913	ENSMUSG00000108258	Chr6:148,915,215-148,920,535	Sinhcaf, Dennd5b, Etfbkmt, Amn1, Resf1, Bicd1, Caprin2, Ipo8, Tmtc1, ERgic2, Far2
Gm36670	ENSMUSG00000109696	Chr8: 91,974,095-91,991,869	Irx5,Irx6, , Mmp2, Lpcat2, Capms2, Slc6a2, Irx3, Fto, Rpgrip1l, Aktip, Rb12, Chc9
Gm39465	ENSMUSG00000111389	Chr9:122,029,509-122,051,508	Snrk, Ano10, Abhd5, Topazi, Tcaim, Zfp445, Zkscan7, Zfp105, Kif15, Tmem42, Tgm4, Pomgnt2, Gask1a, Ackr2, Cyp8b1, Higd1a, Ccdc13, Klh40, Hhat1, Nktr, Ss1812, Zfp651, Sec22c, Vipr1, Lyz14, Cck, Trak1, Ulk4
Gm43332	ENSMUSG00000107313	Chr5:149,183,183-149,184,226	Uspli, Alox5ap, Medag, TEx26, Wdr95, Hsph1, B3glct, Rxfp2, Hmgb1, Katnali, Ubl3, Slc7a1, Mtus2
2900052N01Rik	ENSMUSG00000099696	Chr9:46,913,603-46,927,366	Cadm1, Apoa5, Bud13, Zpr1, Apoa4, Apoc3, Apoa1, Pafah1b2, Sidt2, Tagln, Pcsk7
Gm36161	ENSMUSG00000114608	Chr13:120,016,880-120,018,215	AF067961, BC147527, Tcstv3, Tcstv1, D13Ertd608e, AF067063, Zfp131, Nim1k, Hmgcs1, Cc128, Tmem267, Paip1, Nnt
Gm16685	ENSMUSG00000097804	Chr3:7,612,705-7,690,001	Stmn2, Hey1, Il7, Zc2hc1a, Pkia
Gm26870	ENSMUSG00000097312	chr9:3,017,875-3,038,310	Alkbh8, Cwf1912, Gucy1a2
Gm15743	ENSMUSG00000086645	Chr16:31,427,876-31,429,776	Bdh1, Dlg1, Meltf, Pigz, Ncbp2, Senp5, Pak2, Pigx, Cep19, Nrros, Bex6, Fbxo45, Wdr53, Smco1, Rnf168, Ubxn7, Tm4sf19, Tctex1d2, Apod, Ppp1r2, Acap2, Xxylt1, Fam43a, Lsg1, Tmem44
2310016D03Rik	ENSMUSG00000102098	Chr12:30,410,559-30,467,358	Tmem18, Alka12, Acp1 Sh3yli, Fam110c, Lamb1, Dld, Slc26a3, Sntg2, Tpo, Pxdn, Myt1l
Gm43913	ENSMUSG00000108258	Chr6:148,915,215-148,920,535	Scinhaf, Dennd5b, Etfbkmt, Amn1, Resf1, Bicd1, Caprin2, Ipo8, Tmtc1, Ergic2, Far2

Supplementary Table 3. Top 50 differentially expressed lncRNAs and neighbouring genes in Harlequin mice.

MGI Symbol	*Ensembl ID*	*Locus*	*Genes (1,000,000bp)*
Xist	ENSMUSG00000086503	chrX:103,460,366-103,483,254	Zcchc13, Slc6a2, Rlim, Nexmif, Abcb7, Tsx, Cdx4, Nap1l2, Dmrtc1a, Dmrt1c2, Dmrtc1b, Phka1
Gm15903	ENSMUSG00000086916	chr5:129,727,046-129,728,385	Psph, Sumf2, Phkg1, Nupr1l, Chchd2, Vkorc1l1, Crpcp, Tpst1, Zbed5, Gusb, Asl, Kctd7, Rabgef1, Tmem248, Sbds, Tyw1, Caln1, Nipsnap2, Mrps17, Sept14, Zfp11, Sfswap, Adrd1, Ran, Stx2, Rimbp2, Piwil1
Gm32171	ENSMUSG00000111645	chr9:33151292-33262516	Ets1, Fli1, Kcnj1, Kcnj5, Arhgap32
2410006H16Rik	ENSMUSG00000086841	chr11:62602877-62604807	Lrrc75a, Mmgt2, Zfp287, Trim16, Fbxw10, Tvp23b, Cdrt4, Tekt3, Pmp22, Trpv2, Cenpv, Ubb, Pigl, Ncor1, Ncor1, Zswim7, Ttc19, Specc1, Akap10, Ulk2, Prpsap2, Grap, Slc5a10, Fam83g
2610035D17Rik	ENSMUSG00000087259	chr11:113043895-113201838	Slc39a11, Sstr2, Cog1, Fam104a, D11Wsu47e, Cpsf4l, Cdc42ep4, Sdk2, Sox9
Gm15564	ENSMUSG00000086324	chr16:35966753-35983230	KPn1, Wdr5b, Ccdc58, Csta1, Fam162a, Stfa2l1, Cstdc4, Csta3, Csta2, Stfa1, Cstdc6, Stfa2, Stfa3, Casr, Cd86, Ildr1, Slc15a2, Eaf2, Golgb1, Igcb1, Hlcs1, Fbxo40, Parp9, Dtx3l, Parp14, Hspbap1, Slc49a4, Sema5b, Pdia5, Sec22a, Adcy5, Hacd2, Mylk
Cep83os	ENSMUSG00000097164	chr10:94671025-94688576	Cep83, Plxnc1, Cradd, Socs2, Mrp142, Ube2n, Nudt4, Anapc15-ps, Tmcc3, Ndufa12, Nr2c1, Fgd6, Vezt, Metap2, Usp44, Ntn4
E230013L22Rik	ENSMUSG00000096957	chr8:11477929-11480241	Naxd, Cars2, Ing1, Ankrd10, Arhgef7, Tex29, Sox1, Rab20, Col4a2, Col4a1, Irs2, Myo16
Gm34030	ENSMUSG00000109697	chr8:57331692-57334387	Scrg1, Sap30, Hmgb2, Galnt7, Galnt16, Hand2, Fbxo8, Cep44

Snhg8	ENSMUSG00000104960	chr3:123507552-123508404	Ndst3, Tram1l1, Prss12, Mettl14, Sec24d, Synpo2, Myoz2, Usp53, Fabp2, Pde5a, Fnbp1l
4933404O12Rik	ENSMUSG00000097908	chr5:136919401-136937112	Fis1, Cldn15, Znhit1, Plod3, Vgf, Ap1s1, Serpine1, Trim56, Muc3, Muc3a, Ache, Ufsp1, Srrt, Trip6, Slc12a9, Ephb4, Xan, Epo, Pop7, Gigyf1, Gnb2, Actl6b, Tfr2, Mospd3, Fbxo24, Pcolce, Sap25, Lrch4, Irs3, Agfg2, Tsc22d4, Nyap1, Ppp1r35, Tsc22d4, Mepce, Zcwpw1, Pilr1, Pilrb1, Ift22, Col26a1, Myl10, Cux1, Sh2b2, Prkrip1, Orai2, Polr2j, Rasa4, Lrdw1, Alkbh4, Upk3bl, Dtx2, Zp3, Ssc4d
Gm17017	ENSMUSG00000090246	chr1:85641222-85650585	Sp100, Cab39, Itm2c, Spata3, Gpr55, Psmd1, Htr2b, Armc9, B3gnt7, Nc1, Nmur1, TEx44, Ptma, Pde6d, Cops7b, Sp140, Sp110, Slc16a14, Fbxo36, Trip12, Dner
Gm7292	ENSMUSG00000104222	chr6:117716342-117718868	Zfp637, Zfp239, Hnrnpf, Fxyd4, RAsgef1a, Csgalnact2, Ret, Bms1, Zfp248, Zfp9, Ankrd26, Cacna1c, Cxcl12
Gm20632	ENSMUSG00000093577	chr3:96239393-96241819	H3c15, H2ac19, H2ac18, H3c14, H4c14, H2bc18, Fcgr1, H3c13, BC107364, Hjv, Txnip, Polgr3gl, Ankrd34a, Rbm8a, Pex11b, Itga10, Ankrd35, Nudt17, Polr3c, Pias3, Rnf115, Cdc60, Pdck1, Gpr89, Gja5, Gja8, Acp6, Bc19, H2ac20, H2ac21, Bola1, Sv2a, Otud7b, Mtmr11, Sf3b4, Otud7b, Vps45, Plekho1, Anp32a, Mrps21, Prpf3, Ciart, Aph1a, Car14, BC028528, Rprd2, Ecm1, Tars2, Ensa, Mcl1, ADamts14, Golph3l, Hormad1, Ctss, Ctsk, Arnt, Setdb1, Cers2, Anxa9, Mindy1, Prun1
Gm16685	ENSMUSG00000097804	chr3:7612705-7690001	Stmn2, Hey1, Il7, Zc2hc1a, Pkia

Gm38534	ENSMUSG00000108634	chr11:88253368-88256990	Ccdc182, Msi2, Akap1, Scep1, Elob1, Coil, TRim25, Dgke, Mrps23, Cuedc1, Vezf1, Srsf1, Dynll2, Olfr464, Olfr462, Olfr463, Epx, Mks1, Lpo. Mpo, Tspoap1, Supt4a, Rnf43, Hsf5, Mtmr4, Sept4, Tex14, Rad51c, Ppm1e
2310015D24Rik	ENSMUSG00000099411	chr16:13514131-13520419	Parn, Bfar, Pla2g10, Rrn3, Ntan1, Pdxdc1, Mpv17l, Ofitm7, Bmerb1, Marf1, Nde1, Myh11, Fopnl, Abcc1, Mrtfb, ERcc4
Eprn	ENSMUSG00000113346	chr13:73344665-73347384	Lpcat1, Slc6a3, Clptm1l, Slc6a18, Tert, Slc12a7, Nkd2, Trip13, Brd9, Zdhhc11, Tppp, Cep72, Slc9a3, Exoc3, Ahrr, Pdcd6, Dsha, Mrp136, Ndufs6, Irx4, Irx2
Gm47101	ENSMUSG00000112073	chr10:102435269-102435543	Nts, Rassf9, Alx1, Lrriq1, Slc6a15, Mgat4c
2900097C17Rik	ENSMUSG00000102869	chr2:156388065-156392979	Epb4l1, Aar2, Dlgap4, Tgif2, Rab5if, Sla2, Ndrg3, Dsn1, Soga1, Tldc2, Samhd1, Rbl1, Mroh8, Rpn2, Ghrh, Cnbd2, Scand1, Phf20, Romo1, Rbm39, Rbm12, Nsf1, Cpne1, Spag4, ERgic3, Fer1l4, Gdf5, Cep250, Uqcc1, Eif6, Mmp24, Edem2, Procr, BC029722, Gss, Acss2, Myh7b, Trpc4ap, Ggt7, Ncoa6
Gm7125	ENSMUSG00000069996	chr11:51898966-51909624	Ube2b, Cdkn2aipnl, Cdk13m Ppp2ca, Olfr1373, Skp1a, Olfr1371, Skp1a, Tcf7, Vdac1, Jade2, Sar1b, Sec24a, N4bp3, Rmnd5b, Nhp2, Hnrnpab, Phykp1, Col23a1, Clk4, BC049762, Zfp354a, Olfr51, Olfr54, Olfr1375, Olfr1377, Olfr1378, Prop1, Zfp354b

Gm39822	ENSMUSG00000110618	chr11:51898966-51909624	Cdkn2aipn1, Ube2b, Cdkl3, Ppp2ca, Olfr1373, Olfr1371, Skp1a, Tcf7, Vdac1, Jade2, Sar1b, Sec24a, N4bp3, Rmnd5b, Nnp2, Hnrnpab, Phykp1, Col23a1, Clk4, BC049762, Zfp354a, Olfr1375, Olfr54, Olfr51, Olfr1377, Olfr1378, Prop1, Zfp354b, Zfp2
4632404M16Rik	ENSMUSG00000106296	chr3:102134615-102137496	Vangl1, Ngf, Tspan2, Tshb, Sycp1, Nr1h5, Sike1, Csde1, Nras, Ampd1, Casq2, Nhlh2, Slc22a15, Mab21l3, Atp1a1, Igsf3, Cd2
Zbtb11os1	ENSMUSG00000102101	chr16:55973268-55974617	Zbtb11, Pcnp, Trmt10c, Senp7, Impg2, Ani3bp, Tfg, Adgrg7, Tmem45a, Lnp1, Rp124, Cep97, Nxpe3, Nfkbiz, Zpld1
Gm35021	ENSMUSG00000109714	chr8:35724382-35734768	Cldn23, Prag1, Lonrf1, Trmt9b, Dlc1, Mfhas1, Eri1, Ppp1r3b, Tnks, Dusp4
Gm46218	ENSMUSG00000110830	chr10:29297934-29313825	Echdc1, Rnf146, Rspo3, Cenpw, Soga3, Themis, Ptprk
D830013O20Rik	ENSMUSG00000056359	chr12:73364075-73409557	Tmem30b, Prkch, Hif1a, Snapc1, Syt16, Trmt5, Slc38a6, Mnat1, Six4, Six1, Six6, Ppm1a, Dhrs7, Pcnx4, Lrrc9
Snhg1	ENSMUSG00000108414	chr19:8723475-8726443	Wdr74, Stx5a, Nxf1, Tmem223, Tmem179b, Taf6l, Polr2g, Ttc9c, Hnrnpul2, Lrrn4cl, Zbtb3, Bscl2, Ubxn1, Lbhd1, Ints5, Ganab, B3gat3, Gng3, Uqcc3, Rom1, Em13, Mta2, Eef1g, Ahnak, Scgb1a1, Asrgl1, Chrm1, Slc3a2, Slc22a8, Slc22a6, Slc22a30, Slc22a29, Slc22a28, Slc22a27, Slc22a26
Dubr	ENSMUSG00000022639	chr16:50719294-50732773	Ccdc54, Bbx, Cd47, Ift57
Rmst	ENSMUSG00000112117	chr10:92075123-92075629	Nedd1, Cfap54, Tmpo, Slc25a3, Ikbip

AI480526	ENSMUSG00000090086	chr5:123134843-123141666	Setd1b, Hpd, Psmd9, Wdr66, Bc17a, Mlxip, Il31, Lrrc43, B3gnt4, Diablo, Vps33a, Clip1, Zcchc8, Rsrc2, Kntc1, Hcar2, Hcar1, Denr, Ccdc62, Hip1r, Vps37b, Abcb9, Ogfod2, Arl6ip4, Rhof, Tmem120b, Morn3, Orai1, Kdm2b, Rnf34, Anapc5, Camkk2, P2rx4, P2rx7, Ift81, Atp2a2, Anapc7, Arpc3, Fam216a, Vps29, Gph3, Rad9b, Pptc7, Tctn1, Hvcn1, Ppp1cc
Gm20633	ENSMUSG00000093553	chr3:96243550-96246380	H2ac18, h3c14, H4ac14, H2bc18, H3c13, Fcgr1, BC107364, Hjv, Txnip, Polr3gl, Ankrd34a, Rbm8a, Pex11b, Itga10, Lic1l, Ankrd35, Nudt17, Polr3c, Pias3, Rnf115, Cd160, Pdzk1, Gpr89, Gja8. Gja5, Acp6, Bc19, H2ac19, H3c15,H2bc21, H2ac20, H2ac21, Bola1, Otud7b, Mtmr11, Sf3b4, Vps45, Plekho1, Anp32e, Aph1a, Car14, Prpf3, Mrps21, Rprd2, Tars2, Ecm1, Ensa, Mcl1, Adamts14, Golph3l, Hormad1, Ctss, Ctsk, Arnt, Setdb1, Anxa9, Mindy1, Prune1
1700030C10Rik	ENSMUSG00000099759	chr12:20804381-20815779	Asap2, Utgb1bp1, Cpsf3, Iah1, Adam17, Ywhaq
Zswim6	ENSMUSG00000032846	chr13:107724618-107890064	Smim15, Ndudaf2, ERcc8, Elovl7, Depdc1b, Pde4d, Kif2a, Dimt1, Ipo11

E330017L17Rik	ENSMUSG00000086369	chr4:129906433-129919787	Spocd1, Adgrb2, Col16a1, Pef1, Hcrtr1, Tinagl1, Serinc2, Ldc1, Fabp3, Zcchc17, Snrnp40, Nkain1, Pum1, Sdc3, Ptp4a2, Khdrbs1, Tmem39b, Kpna6, Txlna, Iqcc, Ccdc28b, Tmem234, Dcdc2b, Eif3i, Lck, Fam167b, Hdac1, Marcksl1, Fam229a, Tssk3, Bsdc1, Zbtb8b, Zbtb8a, Zbtb8os, Rbbp4, Sync, C77080, Yars, S100pbp, Fndc5, Hpca, Rnf19b, Tmem54, Ak2, Azin2
Gm43244	ENSMUSG00000105342	chr3:102129938-102131222	Vangl1, Ngf, Tspan2, Tshb, Sycp1, Nr1h5, Sike1, Csde1, Nras, Ampd1, Dennd2c, Casq2, Nhlh2, Slc22a15, Mab21l3, Atp1a1, Igsf3, Cd2
Kcnq1ot1	ENSMUSG00000101609	chr7:143203458-143296549	Cdkn1c, Slc22a18, Phlda2, Nap1l4, Cars, Tnfrsf22, Tnfrsf23, Tnfrsf26, Osbp15, Mrgprg, Mrgpre, Nadsyn, Dhcr7, Acte1, Shank2, Kcnq1, Trpm5, Tssc4, Tspan32, Cd81, Ascl2, Th, Ins2, Igf2, Mrp123, Tnnt3, Lsp1, Prr33, Tnni2, Syt8, Ctsd, Ifitm10, Krtap5-4, Krtap5-1, Krtap5-5
Gm13166	ENSMUSG00000085525	chr4:146251829-146253858	Zfp992, Zfp981, Zfp993, Zfp989, Rex2, Zfp991, Zfp600, Zfp987, Zfp986, Zfp980, Zfp990, Tnfrs8
Gm28875	ENSMUSG00000100975	chr12:104925501-104926689	Syne3, Glrx5, Tcl1b2, Tcl1b1, Tcl1b5, Tcl1b4, Tcl1b3, Tcl1, Tunar, Bdkrb2, Bdkrb1, Atg2b, Gskip, Ak7, Papola, Clmn, Dicer1, Gsc, Serpina3n, Serpina3m, Serpina3j, Seroina3k, Serpina3i, Serpina3g, Serpina3f, Serpina3c, Serpina3b, Serpina3a, Serpina5, Serpina12, Serpina9, Serpina11, Serpina1e

2410022M11Rik	ENSMUSG00000100017	chr14:56811989-56813899	Zmym2, Gja3, Gjb2, Gjb6, Cryl1, Ift88, Eef1akmt1, Il17d, Xpo4, Lats2, Sap18, Zmym5, Pspc1, Mphosph8, Parp4, Cenpj, Rnf17, Atp12a, Gzmb, Gzmc, Gzmf, Gzmg, Gzmn, Gzmd, Gzme, Ctsg, Mc[t8, Mcpt4, Mcpt2, Mcpt9, Mcpt1, , Cma2, Cma1, Sdr39u1, Khnyn, Cbln3, Nynrin, Nfatc4
Gm26795	ENSMUSG00000097776	chr8:28593505-28595599	Unc5d
A930029G22Rik	ENSMUSG00000096988	chr17:69416660-69439304	Akain1, Dlgap1, Zbtb14, Epb413, Tmem200c, L3mbtl4
Snhg12	ENSMUSG00000086290	chr4:132308678-132311024	Trnau1ap, Phactr4, Rxx1, Med18, Sesn2, Atpif1, Ptafr, Dnajc8, Eya3, Xkr8, Smpdl3b, Rpa2, Themis2, Ppp1r8, Stx12, Fam76a, Fgr, Ahdc1, Wasf2, Gpr3, Cd164l2, Map3k6, Sytl1, Tmem222, Rab42, Taf12, Gmeb1, Ythdf2, Oprd1, Epb41, Tmem200b, Srsf4, Mecr, Ptbru
Gm5532	ENSMUSG00000073535	chr1:155520173-155527111	Acbd6, Lhx4, Qsox1, Cep350, Tor1aip1, Tor1aip2, Fam163a, Tdrd5, Mphs2, Axdnd1, Soat1, Xpr1, BC034090, Stx9, Mr1, Oer5, Cacna1e

Gm17251	ENSMUSG00000090952	chr17:33759992-33761870	Angptl4, Kank3, Rps28, Kifc1, Ndufa7, Smim40-ps, Cd320, Smim40, Daxx, Zbtb22, Tapbp, B3galt4, Rps18, Vps52, Rgl2, Pfdn6, WDr46, H2-K1, Ring1, Rxrb, H2-Ke6, Slc39a7, Col11a2, Brd2, H2-Dma, H2-DMb2, H2-DMb1, Psm9, Tap1, Psmb8, H2-Ob, Tap2, H2-Ab1, H2-Aa, H2-Ea-ps, Btnl2, H2-Eb1, H2-Eb2, Btnl1, BC051142, Btn14, Btn16, Notch4, Gpsm3, Ppt2, Pbx2, Ager, Rnf5, Agpat1, Egf18, Prrt1, Fkbpl, Atf6b, Tnxb, C4b, Rab11b, March2, Rab11b, Hnrnpm, Zfp414, Pram1, Myo1f, ADamts10, Act19, Zfp101, Zfp955b, Olfr63, Zfp955a, Olfr1564, Olfr239, Olfr55, Morc2b, Zfp563, Zfp763, Zfp952, , Zfp472, Cyp4f13, Cyp4f14, Zfp870, Zfp799, Zfp811, Zfp871
Ifi30	ENSMUSG00000031838	chr8:70762774-70766663	Pik3r2, Mast3, Ili2rb1, Arrdc2, Kcnn1, Slc5a5, Rpli8a, Map1s, Rpli8a, Ccdc124, Haus8, Myo9b, Use1, Ocel1, Nr2f6, Ushbp1, Babam1, Ankle1, dda1, Ano8, Gtpbp3, Plvap, Ccdc194, Bst2, Mvb12a, Tmem221, Nxnl1, Slc27a1, Fam129c, Colgalt1, Pgls, Unc13a, Jak3, Insl3, B3gnt3, Fcho1, Mpv17l2, Rab3a, Pde4c, Lsm4, Gm3336, Pgpep1, Pde4c, Iqcn, Jund, Ssbp4, Gdf15, Lrrc25, Isyna, Fkbp8, Kxd1, uba52, REx1bd, Tmem59l, Crlf1, Klhl26, Crtc1, Comp, Upf1, Gdf1, Cers1, Cope, Ddx49, Sugp2, Homer3, Armc6, Slc25a42, Tmem161a, Mef2b, Borcs8, Rfxank, Nr2c2ap, Ncan, Hapln4, Tm6sf2, Sugp1, Mau2, Gatad2a, Tssk6, Cilp2, Pbx4, Yjefn3, Ndufa13, Gatad2a, Atp13a1, Gmip, Lpar2

Gm47547	ENSMUSG00000114196	chr14:41053653-41061614	Mat1a, Mbl1, Sftpd, Sftpd1, Gm2832, Gm5798, Gm7945, Gm6482, Gm7954, Gm3486, Gm7970, Gm3072, Gm3676, Gm8068, Gm7929, Gm47189, Gm7980, Gm6401, Gm3543, Dydc1, dydc2, Prxl2a, Tspan14, Sh2d4b
Gm20300	ENSMUSG00000111080	chr10:30603195-30606634	Hint3, Ncoa7, Hey2, Hddc2, Tpd52l1, Rnf217, Trmt11, Cenpw
Gm47469	ENSMUSG00000113047	chr13:74553910-74556948	Erap1, Cast, Pcsk1, Zfp825, Ftl1-ps1, Zfp72, Lrrc14b, Ccdc127, Sdha, Pdcd6, Ahrr, Exoc3, Slc9a3, Cep72, Tppp, Zdhhc11, Brd9, Trip13, Nkd2, Slc12a7, Slc6a19, Slc6a18, Tert, Clptm1l
